# The Role of Innate Lymphoid Cells in Chronic Respiratory Diseases

**DOI:** 10.3389/fimmu.2021.733324

**Published:** 2021-09-22

**Authors:** Amy T. Hsu, Timothy A. Gottschalk, Evelyn Tsantikos, Margaret L. Hibbs

**Affiliations:** Department of Immunology and Pathology, Central Clinical School, Monash University, Melbourne, VIC, Australia

**Keywords:** pulmonary inflammation, airway inflammation, obstructive lung disease, COPD, asthma, NK cells, innate lymphoid cells (ILC)

## Abstract

The lung is a vital mucosal organ that is constantly exposed to the external environment, and as such, its defenses are continuously under threat. The pulmonary immune system has evolved to sense and respond to these danger signals while remaining silent to innocuous aeroantigens. The origin of the defense system is the respiratory epithelium, which responds rapidly to insults by the production of an array of mediators that initiate protection by directly killing microbes, activating tissue-resident immune cells and recruiting leukocytes from the blood. At the steady-state, the lung comprises a large collection of leukocytes, amongst which are specialized cells of lymphoid origin known as innate lymphoid cells (ILCs). ILCs are divided into three major helper-like subsets, ILC1, ILC2 and ILC3, which are considered the innate counterparts of type 1, 2 and 17 T helper cells, respectively, in addition to natural killer cells and lymphoid tissue inducer cells. Although ILCs represent a small fraction of the pulmonary immune system, they play an important role in early responses to pathogens and facilitate the acquisition of adaptive immunity. However, it is now also emerging that these cells are active participants in the development of chronic lung diseases. In this mini-review, we provide an update on our current understanding of the role of ILCs and their regulation in the lung. We summarise how these cells and their mediators initiate, sustain and potentially control pulmonary inflammation, and their contribution to the respiratory diseases chronic obstructive pulmonary disease (COPD) and asthma.

## Chronic Inflammatory Lung Diseases Are an Escalating Global Health Issue

COPD is an irreversible chronic inflammatory lung disease that is the third leading cause of death worldwide (1). Patients with COPD exhibit airflow limitation, progressive deterioration in lung function and experience exacerbations; an acute worsening of their symptoms, often driven by lung infection (2). The major risk factor for COPD is cigarette smoking, although other risks such as environmental pollution or premature birth increasingly contribute to COPD susceptibility (3). COPD is underpinned by chronic inflammation, resulting in lung pathologies such as emphysema due to alveolar tissue destruction, and chronic bronchitis arising from goblet cell metaplasia and mucus overproduction (4). Inducible bronchus-associated lymphoid tissue (iBALT) often develops in COPD, particularly in advanced disease (5). COPD is heterogeneous and various disease processes, inflammatory cells (macrophages, neutrophils, cytotoxic T cells, T helper (Th)-1/17 cells) and cytokines are involved (6).

Asthma is a mostly reversible inflammatory airway disease affecting around 300 million people worldwide, where exaggerated swelling and narrowing of the conducting airways (airway hyperresponsiveness; AHR) is triggered in susceptible individuals by the inhalation of environmental particles (7–9). Asthma is differentiated into subtypes―allergic or non-allergic, and by severity―mild-intermittent, mild, moderate, or severe or by the dominant inflammatory response―eosinophilic or neutrophilic. The most common type of asthma is allergic or eosinophilic asthma, which is characterised by a type 2 immune response (driven by cytokines IL-4, IL-5 and IL-13) and IgE-mediated hypersensitivity (10). Conversely, during non-type 2 asthma, neutrophils, alongside a Th1/Th17 skewed response, predominate (11, 12). Severe asthma, which is predominantly neutrophilic, affects 5-10% of patients and is often unresponsive to standard corticosteroid-based therapies (13, 14). Asthma-COPD overlap (ACO) is a syndrome where patients exhibit characteristics of both asthma and COPD (15), complicating the study of inflammatory lung diseases.

This review will focus on ILCs and their involvement in COPD and asthma.

## The Emergence of ILCs in Immunity

ILCs are a somewhat newly identified family of innate immune cells that have garnered intense recent attention and our understanding of their biological roles is rapidly progressing. ILCs are mainly tissue-resident (16) and enriched at mucosal sites such as the respiratory, gastrointestinal and reproductive tracts, where they act as first responders to pathogens, aiding the innate immune system to launch a rapid defence, in addition to having roles in tissue repair and homeostasis (17). ILCs closely resemble Th cells in their development and function (18). They lack conventional antigen receptors, instead recognising non-specific danger signals, microbial compounds and cytokines (18), yet can also develop immunological memory (19). While ILCs and T cells have overlapping functions, ILCs perform additional non-redundant roles in priming adaptive immune responses (20). Like their T cell counterparts, ILCs are implicated in chronic inflammation, autoimmunity, and cancer (21–23).

The ILC family comprises five main subsets, which include natural killer (NK) cells, lymphoid tissue inducer (LTi) cells (which play a key role in the development of lymphoid tissues), ILC1, ILC2 and ILC3. ILCs have characteristics and functions that resemble adaptive CD4^+^ Th cell subsets. ILCs are classified into three main groups: group 1 (ILC1 and NK cells), group 2 (ILC2) and group 3 (ILC3 and LTi cells), which correspond to Th1 (NK cells correspond to CD8+ cytotoxic T cells), Th2 and Th17 cells respectively (24), based on similar transcription factors and functional profiles (25–27). ILCs derive from the common lymphoid progenitor and primarily develop in the foetal liver or in the bone marrow after birth (28). ILC1, ILC2 and ILC3, but not conventional NK cells, develop from Id2^+^ common helper-like innate lymphoid precursor cells (29), whereas conventional NK cells likely branch off earlier in development (30). Tissue-resident ILCs can be replenished from bone marrow or lymphoid organ precursors however, they are predominantly maintained through local self-renewal and expansion at tissue sites (16). While little is known about how ILC1s populate the lung, ILC2s and ILC3s arise in the lung shortly after birth, with ILC2 seeding dependent on production of IL-33 by type II alveolar epithelial cells (31) and ILC3s on insulin-like growth factor 1 from alveolar fibroblasts (32). ILCs are lineage-negative, lacking common lymphoid and myeloid lineage markers, and this feature is used to distinguish them by flow cytometry. ILCs are highly plastic and can change their phenotype and function depending on environmental signals, and their identification can also be complicated by their maturity (33).

## Phenotypic Features of ILC Subsets

ILC1s and NK cells require the transcription factor T-bet for their development; however, NK cells additionally utilise Eomes (34). ILC1s and NK cells secrete interferon-gamma (IFN-γ) and tumour necrosis factor alpha which are key in the defence against intracellular pathogens. NK cells employ both a cytotoxic (CD56^dim^ subset) and cytokine (CD56^bright^ subset) response (35, 36). Both ILC2s and ILC3s have the potential to differentiate into ILC1 or ILC1-like cells (37, 38). Indeed, STAT-1, a key transcription factor activated during bacterial and viral infections, has been found to skew the differentiation of ILCs toward ILC1 while suppressing ILC2 and ILC3 responses (39).

ILC2s are dependent on the transcription factor GATA-3 and support Th2 immune responses *via* production of type 2 cytokines such as IL-4, IL-5, and IL-13 (40), which are essential for defence against extracellular parasites but can also drive allergic responses. ILC2s are the predominant ILC subset in the steady-state lung, where they secrete amphiregulin to promote pulmonary wound healing after infection, suggesting a homeostatic function (41). In mice, two distinct ILC2 populations have been characterized: natural ILC2s that are identified as Lineage^-^ST2^+^KLRG1^int^ and classified as homeostatic, tissue-resident and IL-33-responsive; and, inflammatory ILC2s, which are undetectable at the steady-state but expand in response to IL-25 and can be distinguished as Lineage^-^ST2^-^KLRG1^hi^ cells (42). ILC2s are activated by IL-33, IL-25, thymic stromal lymphopoietin (TSLP) and other danger signals produced by the airway epithelium (43, 44), with further support from prostaglandin D_2_ signalling through the CRTH2 receptor (40). Additionally, p38 MAPK has been found to positively regulate ILC2 function (45) while TGF-β is thought to program development *via* induction of ST2 expression in ILC2 progenitors (46). IL-1β is critical for ILC2 plasticity by inducing T-bet expression and promoting conversion into ILC1s in response to the Th1 cytokine IL-12 (47).

ILC3s and LTi cells require the transcription factor RORγt for their induction, and generate Th17-like responses, producing the cytokines IL-17, IL-22, and GM-CSF (24, 48). LTi cells also play an important role in lymphoid organogenesis in foetal development (49–51). ILC3s can be further sub-grouped by the expression of Natural Cytotoxicity Receptors (NCRs) such as NKp46 (NCR^-^ or NCR^+^) (52, 53); while the expression of CCR6 and T-bet distinguishes effector cytokine profile (NCR^-^CCR6^+^T-bet^-^ produce IL-17, NCR^+^CCR6^-^T-bet^+^ produce IFN-γ) (54). IL-18 can induce ILC3 proliferation and IL-22 production through NF-κB (55), while RANKL expression on ILC3s negatively regulates ILC3 cytokine production (56). Interestingly, the Th2 transcription factor GATA-3 is also critical for the induction and maintenance of ILC3s (57, 58) and therefore unsurprisingly, ILC2s have the potential to differentiate into IL-17-producing ILC3-like cells (59–61).

## ILC Subsets in COPD and Asthma

### ILC1 and NK Cells

#### ILC1 and NK Cells Are Indicators of COPD Severity

**Figure 1:**
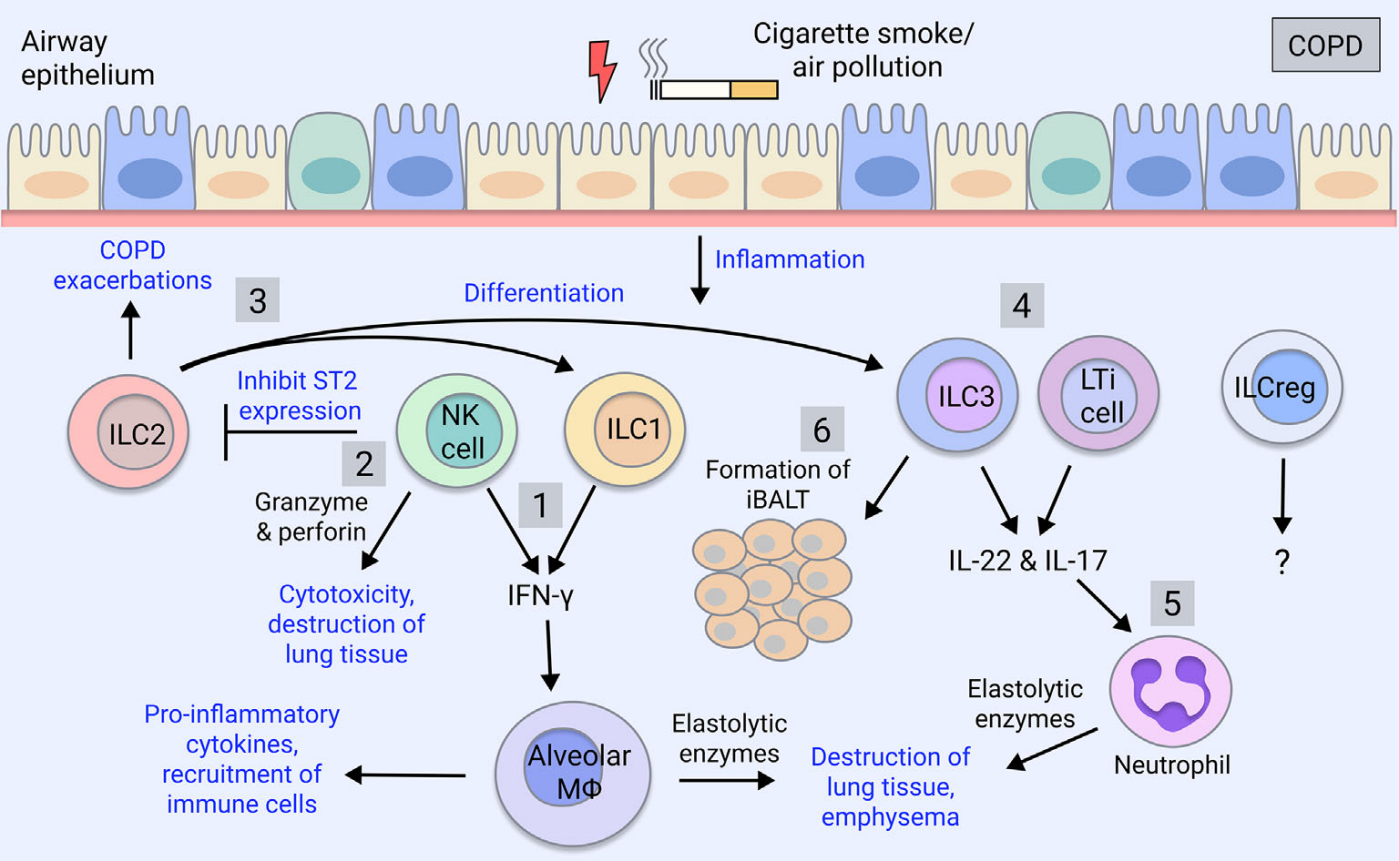
ILC involvement in COPD. COPD is caused by cigarette smoking and insults such as air pollutants. COPD patients exhibit increases in group 1 and group 3 ILCs, which correlate with severity and exacerbations, whereas ILC2 numbers are reduced (63). 1) ILC1 and NK cells produce the pro-inflammatory cytokine IFN-γ, which activates alveolar macrophages causing the release of inflammatory mediators (67). Macrophages secrete proteases (MMPs, cathepsins) inducing the destruction of the lung parenchyma thereby contributing to emphysema (67). 2) NK cell cytotoxic activity through secretion of granzyme and perforin induces death of lung tissue, furthering emphysema (68). NK cells also inhibit the production of ILC2 through downregulation of their ST2 receptor (69). 3) ILC2s promote Th2 inflammation during COPD exacerbations or differentiate into ILC1-like cells in the presence of IL-1β and IL-12 during lung inflammation (37, 63, 70). They potentially also differentiate into ILC3s (59–61). 4) ILC3 and LTi cells produce IL-17 and IL-22, which are elevated in COPD patients, driving pathogenesis (71, 72). 5) IL-17 induces the maturation and recruitment of neutrophils, which are expanded in COPD patients, and *via* their release of proteases (neutrophil elastase, cathepsin G, proteinase-3), contribute to mucus secretion and alveolar destruction (6). 6) ILC3 and LTi cells contribute to the formation of iBALT, which is a feature of advanced COPD (5) and is the site of ILC localisation in COPD lungs (62). ILC_regs_ are yet to be understood in the regulation of COPD pathogenesis.

Recent studies suggest that an increased frequency of ILC1s in the peripheral blood of COPD patients correlates with disease severity and increased exacerbation risk (37, 62), and therefore may be utilised as a biomarker for disease progression. Furthermore, ILC1s as well as ILC3s are expanded in the lung of severe COPD patients (63). ILCs tend to localise to lymphoid aggregates in the lungs of COPD patients and smokers, whereas they are found in the parenchyma in healthy individuals (62). Cigarette smoke induces pulmonary ILC1s in a mouse model of COPD (62). ILC1s, alongside Th1 and CD8+ T cells can produce IFN-γ which is implicated in COPD pathogenesis by inducing elastolytic proteases and nitric oxide production by alveolar macrophages, leading to emphysema (64–66) (**Figure 1**). Furthermore, human ILC2s exhibit plasticity *in vitro* as well as *in vivo* when transferred to humanised mice, where they differentiate into ILC1s in the presence of IL-1β and IL-12 during pulmonary inflammation (63) and this is implicated in COPD exacerbations (37) (**Figure 1**).

On the other hand, NK cell-mediated destruction of lung tissue is implicated in COPD as NK cell cytotoxicity is enhanced in the lung of COPD patients, correlating with worsened lung function and emphysema (68) (**Figure 1**). Lung dendritic cells, *via* IL-15Rα signalling, prime NK cell cytotoxicity in the COPD lung, which may represent a therapeutic target (73). NK cell cytokine production is also implicated; in mice, cigarette smoke triggers NK cell pro-inflammatory cytokine release (74) by promoting their expression of the IL-33 receptor, ST2, while inhibiting type 2 responses through downregulating ILC2 expression of ST2 (69) (**Figure 1**). Therefore, NK cells likely contribute to lung emphysematous destruction and inflammation in COPD.

#### NK Cells Have an Ambiguous Role in Asthma

**Figure 2:**
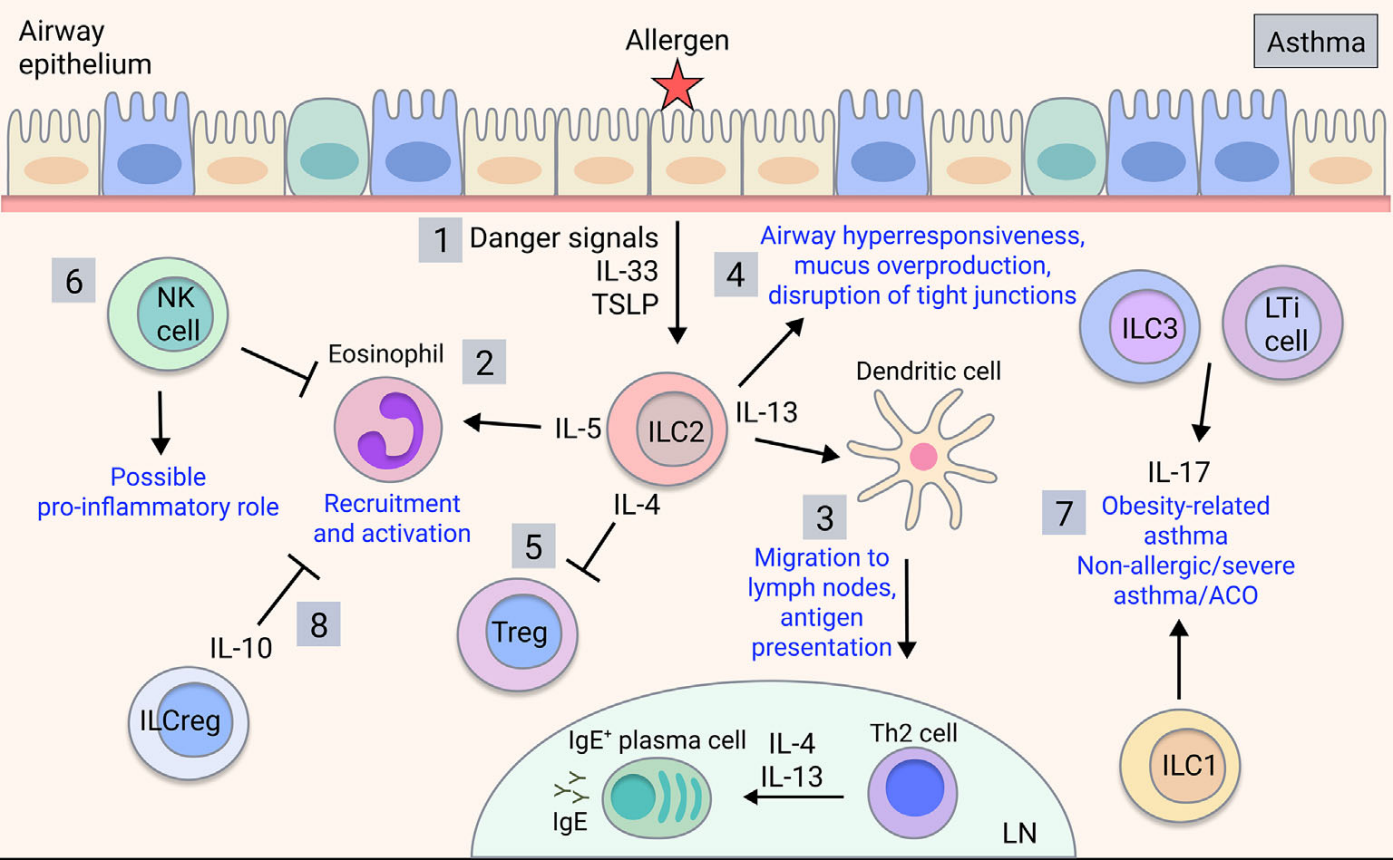
ILC involvement in asthma. Upon allergen detection by airway epithelium, 1) ILC2s are activated by signals released by the airway epithelial cells and other activated immune cells, producing type 2 cytokines such as IL-4, IL-5, and IL-13 in allergic asthma. 2) IL-5 is key for eosinophil recruitment and activation in the lung (77) and 3) IL-13 mediates dendritic cell migration to the lymph nodes, promoting T cell differentiation into effector Th2 cells, which mediate B cell class-switching and IgE production (78). 4) ILC2-derived IL-13 also acts on the airway epithelium to induce airway hyperresponsiveness, mucus overproduction and disruption of barrier integrity (43, 79, 80). 5) ILC2-derived IL-4 may potentially inhibit T_reg_ production in asthma (81). 6) NK cells play an ambiguous role in asthma with both disease-driving and disease-modulatory activity shown. 7) ILC3s/LTi cells and possibly ILC1s contribute to obesity-related asthma and potentially non-allergic, severe asthma or ACO through production of IL-17 (82). 8) ILC_regs_ may regulate asthma by inhibiting eosinophil recruitment through IL-10 (83).

While the contributions of ILC1s in asthma are currently unknown, ILC1s may be relevant to neutrophilic asthma or ACO, which warrants investigation. Meanwhile, NK cells can be both beneficial and detrimental in allergic and severe asthma. NK cells promote resolution of inflammation *via* inducing apoptosis of eosinophils and protecting against viral-induced inflammation (75, 76) (**Figure 2**). Furthermore, the immunomodulatory role of NK cells is impaired in severe asthma, with NK cells showing reduced lipotoxin A_4_-mediated clearance of eosinophils (84). Additionally, NK cell-mediated eosinophil clearance is inhibited by corticosteroids, implicating the loss of NK cell cytotoxicity in severe and steroid-resistant asthma (85). Conversely, NK cells can drive asthma-like allergic airway inflammation by inducing type 2 cytokine production (86–90). However, NK cells played neither a positive nor negative regulatory role in a house dust mite (HDM) model (91). Given that most human studies implicate immunomodulatory rather than pro-inflammatory NK cell functions, it is speculated that this may not be directly recapitulated in mouse models, so further clarification is needed.

### ILC2s

#### ILC2 Involvement in COPD and Exacerbations

ILC2s can convert to ILC1s in the setting of COPD, suggesting skewing towards type 1 inflammation in this disease (37) (**Figure 1**). However, ILC2s themselves have also been implicated in COPD by promoting type 2 inflammatory responses (92), although it is unclear if ACO patients, who exhibit an intermediate type 2 cytokine profile (93), were included in this cohort. Interestingly, ILC2s have been shown to mediate neutrophil recruitment in a model of cigarette smoke-induced COPD and their deficiency protected against emphysema yet promoted fibrosis through elevation of IL-13 and IL-33 (94). Furthermore, ILC2s have been implicated in promoting Th2 adaptive responses during acute COPD exacerbations (70) (**Figure 1**).

#### ILC2s Are Major Players in Allergic Asthma

In allergic asthma, which is commonly associated with type 2 inflammation, there are increases in ILC2s in the peripheral blood compared to healthy individuals or those with allergic rhinitis (95–97), and ILC2s are expanded in the lung of patients with severe asthma and associated eosinophilia (84, 98). In sputum analyses of eosinophilic asthma patients, ILC2s are strongly induced alongside alternatively-activated ‘M2’ macrophages, whereas numbers of alveolar macrophages are unchanged (99). Meanwhile, neutrophilic asthma patients exhibit increases in ILC1s and ILC3s along with inflammatory ‘M1’ macrophages (99) (**Figure 2**). ILC2s are critical in allergic airway inflammation, driving pathology alongside and independent of the adaptive immune system (100). Eosinophil recruitment is induced by ILC2 production of IL-5 (77) whereas their production of IL-13 triggers AHR, mucus overproduction and disruption of epithelial integrity (43, 79, 80) (**Figure 2**). ILC2-derived IL-13 also promotes dendritic cell migration and subsequent Th2 cell induction (78). ILC2 production of IL-4 blocks T_reg_ induction in food allergy responses (81), however this has yet to be shown in asthma. Interestingly, a subset of CCR10^+^ ILC2s that exhibit ILC1-like characteristics and have the capacity to produce IFN-γ, were protective in both allergic and non-allergic severe asthmatic patients by limiting Th2 cytokine secretion and downregulating type 2 responses (101), demonstrating a protective function.

Numerous studies have explored the mechanisms of ILC2 regulation in allergic asthma. The neuropeptide neuromedin U, has been found to powerfully induce an asthma-like response and activate ILC2s (102), suggesting involvement of the neuroimmune axis. IL-1β, arginase 1, lipotoxin A_4_, maresin 1, Nrf2, FABP5, IL-35, IFN-γ and PD-1 have all been shown to restrict ILC2 responses and promote resolution of allergic lung inflammation or prevent AHR through various mechanisms (79, 103–109). Furthermore, in allergic asthma, ILC2s are controlled by the transcription factor IRF7, a key regulator of anti-viral responses (110). On the other hand, neutrophils are reported to control allergic airway inflammation in a mouse HDM asthma model through the inhibition of ILC2 responses and G-CSF modulation (111). Collectively, this suggests that ILC2s may be a viable therapeutic target in asthma.

### ILC3 and LTi Cells

#### Increased Il-17 Levels Implicate Group 3 ILCs in COPD Pathogenesis

In COPD, proportions of lung ILC subsets are skewed toward NCR^-^ ILC3s/LTi cells whereas non-COPD individuals have balanced proportions, with ILC2s and NCR^-^ ILC3s in greatest abundance (112) (**Figure 1**). NCR^-^ ILC3s are also enriched in severe COPD patients (63). In COPD patients and smokers, a highly migratory subset of ILC3s expressing neuropilin-1 receptor is found within the lung and associates with iBALT *via* induction of ICAM-1 and VCAM-1 on mesenchymal stromal cells (113) (**Figure 1**). In mice, ILC3s and ILC1s are increased in response to cigarette smoke exposure, whereas ILC2s are diminished (94). ILC3s are early producers of IL-17 and IL-22, which are implicated in COPD pathogenesis. IL-17 is elevated in the peripheral blood of COPD patients (114, 115) and steroid-resistant COPD (116), and COPD exacerbations have been associated with IL-17 and neutrophilic infiltration (71). IL-22 meanwhile, is elevated in COPD patient lungs and contributes to experimental COPD (72). Additionally, improvements in lung outcomes and comorbidities have been observed in cigarette smoke-exposed IL-17-deficient mice (117, 118). Given the association between ILC3 and IL-17 production, it is surprising that this link has not been addressed in COPD but could be a promising therapeutic avenue.

#### Group 3 ILCs May Be a Player in Neutrophilic Asthma and ACO

Currently, there are limited reports on ILC3s and LTi cells in asthma, however, ILC3-mediated production of IL-17 may contribute (**Figure 2**). IL-17 is implicated in severe asthma, neutrophilic asthma, asthma exacerbations and airway remodelling involving the recruitment of neutrophils (119, 120) and may contribute to ACO given that IL-17 levels are increased in ACO patients (121). Furthermore, the development of AHR in obesity-related asthma in mice relied on IL-17-producing ILC3s and the NLRP3 inflammasome, and ILC3s were expanded in the BAL of severe asthma patients (82). Therefore, *via* IL-17, ILC3s have a potential role in distinct asthma endotypes, although further studies are warranted in human disease.

### ILCregs

#### Regulatory ILCs and Anti-Inflammatory Cytokine Production

Recently, a novel group of regulatory ILCs (ILC_reg_) have been identified in gut, characterised by expression of the immunoregulatory cytokines IL-10 and TGF-β (122). While ILC_regs_ share mechanistic similarities with regulatory T cells (T_reg_), they exhibit a unique transcriptome profile and lack the typical T_reg_ transcription factor FoxP3 (122). Furthermore, ILC_regs_ are transcriptionally distinct from typical ILCs, lacking common ILC transcription factors and can be distinguished as Lineage^-^CD45^+^CD127^+^ cells and by production of IL-10 (122). Moreover, recent reports show that *ex vivo* human ILC1s and ILC2s can also potently produce IL-10 (123). In mice, an induced ILC_reg_ population derived from ILC2s, likely in response to retinoic acid, was observed in the lungs in HDM-induced allergic airway inflammation, with a similar population of cells also detected in human airway tissue (124). Likewise, a subset of ILC2s that produces IL-10 (ILC2_10_) has been directly implicated in decreasing eosinophil recruitment to the injured lung (83) (**Figure 2**). While there is evidence that immunoregulatory ILC subsets are implicated in asthma, this has yet to be reported in COPD.

## The Effects of Therapies on ILC Responses

Recent guidelines recommend that adults with asthma receive a combination therapy comprising inhaled corticosteroids and long-acting β-agonists (125), and for severe asthma, inclusion of biologicals targeting type 2 responses (126). In COPD, while corticosteroids are ineffective, the recommendation for COPD exacerbations is a triple inhaled therapy containing corticosteroids, long-acting β_2_-agonists, and long-acting muscarinic antagonists (127). Studies are beginning to reveal how these agents regulate ILC activity.

Dexamethasone is reported to inhibit type 2 cytokine production from ILC2s (128–131); however, IL-7 and TSLP induce resistance *via* IL-7Rα and STAT5 (129). Conversion of resting CD45RA+ ILC2s to inflammatory CD45RO+ ILC2s is suppressed by corticosteroids; however, once present, inflammatory ILC2s are resistant (132). Furthermore, inflammatory ILC2s are increased in the lung and blood of patients with chronic asthma correlating with disease severity and corticosteroid-resistance (132). In paediatric patients with severe therapy-resistant asthma, ILC2s, eosinophils and Th2 cells are increased, whereas Th17 cells and IL-17+ ILCs are unchanged. Systemic but not inhaled corticosteroids reduced ILC2s and Th2 cells as well as symptoms, despite persistence of IL-17+ cells and eosinophils (133). Collectively these studies suggest that ILC2 activity can be controlled by steroid therapy, but the inflammatory environment may alter steroid responsiveness. Anti-IL-5Rα therapy in patients with severe steroid-dependent asthma reduced blood and sputum eosinophils and IL-5Rα+ ILC2 but not total ILC2. While the functional relevance of IL-5Rα+ ILC2 is unclear, these changes were associated with improved asthma control and lung function (134).

ILC2s express the gene encoding β2-adrenergic receptor, with deficiency of this gene in mice inducing ILC2s and inflammatory responses (135). Furthermore, IL-33-induced ILC2 expansion and IL-5 and IL-13 production in lung were reduced by β2-agonist treatment, suggesting that β2-adrenergic receptor signalling limits the proliferation and function of ILC2s (135). With respect to cholinergic pathways, neuromedin U strongly activates ILC2s and amplifies IL-25-dependent lung inflammation (102, 136, 137). Moreover, the NMUR1 neuromedin U receptor is expressed on ILC2 and its deficiency attenuates ILC2 number and function in allergic airway inflammation (102). While little is known of the effects of muscarinic antagonists on ILCs or whether they express the receptors, in a papain-induced model of airway inflammation, the long-acting muscarinic antagonist tiotropium indirectly suppressed ILC2 activation by reducing IL-4 production from basophils (138). Clearly more studies are required to determine how therapies affect ILC phenotype and function, with these likely providing new insights into the regulation of these cells in chronic lung inflammation.

## Conclusion

Innate lymphoid cells are an incompletely understood accomplice in the maladapted inflammatory environment that promotes chronic respiratory diseases. They potentially play a significant role in disease pathogenesis given their rapid response to pathogenic or environmental stimuli and may be the missing gap where conventional cell-based therapies have failed. However, the identification of ILCs is complex, making their study difficult, and their phenotype may be further complicated by their plasticity, microenvironment, and stages of differentiation. Although ILC manipulation in chronic respiratory diseases represents a significant challenge, understanding the intricacy of ILC regulation and the signals used by other tissue-resident cells to control their responses may provide a means of targeting them indirectly. The emergence of regulatory ILCs suggests another level of disease control, however these cells may be impaired or overwhelmed in chronic inflammatory settings. Nonetheless, ILCs represent a developing field in disease research. Understanding the role of ILCs during chronic inflammatory airway diseases like COPD and asthma will provide insight into the complexity of these diseases, how they are initiated and the manner in which they transition into a chronic state. Additionally, understanding how ILCs are regulated, how they respond to conventional treatments and furthermore how they regulate immune responses may allow us to devise strategies to switch their disease-driving capabilities into disease-modulatory actions.

## Author Contributions

MH conceptualised the article, contributed to the writing of the first draft and provided manuscript revisions. AH wrote the first draft, revised the manuscript and developed original figures. TG and ET amended the manuscript and provided extensive editorial input. All authors contributed to the article and approved the submitted version.

## Funding

This work was supported by funding awarded to MH from the National Health and Medical Research Council (NHMRC) grant number 1141208. AH is the recipient of an Australian Government Research Training Program (RTP) Scholarship.

## Conflict of Interest

The authors declare that the research was conducted in the absence of any commercial or financial relationships that could be construed as a potential conflict of interest.

## Publisher’s Note

All claims expressed in this article are solely those of the authors and do not necessarily represent those of their affiliated organizations, or those of the publisher, the editors and the reviewers. Any product that may be evaluated in this article, or claim that may be made by its manufacturer, is not guaranteed or endorsed by the publisher.
